# Behavioural and Physiological Responses of *Gammarus pulex* Exposed to Cadmium and Arsenate at Three Temperatures: Individual and Combined Effects

**DOI:** 10.1371/journal.pone.0039153

**Published:** 2012-06-22

**Authors:** Céline Vellinger, Vincent Felten, Pascal Sornom, Philippe Rousselle, Jean-Nicolas Beisel, Philippe Usseglio-Polatera

**Affiliations:** Laboratoire des Interactions, Ecotoxicologie, Biodiversité, Ecosystèmes (LIEBE), CNRS UMR 7146, Université de Lorraine, Metz, France; Institut Pluridisciplinaire Hubert Curien, France

## Abstract

This study aimed at investigating both the individual and combined effects of cadmium (Cd) and arsenate (AsV) on the physiology and behaviour of the Crustacean *Gammarus pulex* at three temperatures (5, 10 and15°C). *G. pulex* was exposed during 96 h to (i) two [Cd] alone, (ii) two [AsV] alone, and (iii) four combinations of [Cd] and [AsV] to obtain a complete factorial plane. After exposure, survival, [AsV] or [Cd] in body tissues, behavioural (ventilatory and locomotor activities) and physiological responses (iono-regulation of [Na^+^] and [Cl^−^] in haemolymph) were examined. The interactive effects (antagonistic, additive or synergistic) of binary mixtures were evaluated for each tested temperature using a predictive model for the theoretically expected interactive effect of chemicals. In single metal exposure, both the internal metal concentration in body tissues and the mortality rate increased along metallic gradient concentration. Cd alone significantly impaired both [Na^+^] and [Cl^−^] while AsV alone had a weak impact only on [Cl^−^]. The behavioural responses of *G. pulex* declined with increasing metal concentration suggesting a reallocation of energy from behavioural responses to maintenance functions. The interaction between AsV and Cd was considered as ‘additive’ for all the tested binary mixtures and temperatures (except for the lowest combination at 10°C considered as “antagonistic”). In binary mixtures, the decrease in both ventilatory and locomotor activities and the decline in haemolymphatic [Cl^−^] were amplified when respectively compared to those observed with the same concentrations of AsV or Cd alone. However, the presence of AsV decreased the haemolymphatic [Na^+^] loss when *G. pulex* was exposed to the lowest Cd concentration. Finally, the observed physiological and behavioural effects (except ventilation) in *G. pulex* exposed to AsV and/or Cd were exacerbated under the highest temperature. The discussion encompasses both the toxicity mechanisms of these metals and their interaction with rising temperature.

## Introduction

Trace metals are natural constituents of rocks and soils but their rising concentration in the environment is directly linked to anthropogenic activities [1], [2]. The increasing trace metal concentration in aquatic ecosystems (waters, sediments) has become a major concern. As metals rarely occur alone, living organisms are generally multi-contaminated. The toxicity of metals in mixtures can be additive, but also significantly more or less important than the toxicity theoretically expected by simple addition of the independent metal effects (i.e. synergistic or antagonistic, respectively; [3]). Cadmium (Cd) is a non-essential metal leading to adverse toxic effects at low concentration (µg L^−1^ order), which are well documented for aquatic organisms [4], [5]. In contrast, far less information is available on arsenic effects, and especially arsenate (AsV), one of the inorganic forms of arsenic frequently found in aquatic environments.

Environmental variables such as temperature can influence metal solubility, and then metal bioavailability, leading to change in metal toxicity within exposed organisms [6]–[8]. Generally, a rising temperature enhances pollution toxicity [9]. Freshwater organisms are vulnerable to temperature change, especially ectothermic organisms that have body temperatures dependent of their environment [10]. Temperature changes can affect not only physiological rates and biochemical reactions but also the biological membrane stability and systemic functions, as ventilation or food absorption [10]–[12]. Temperature also modifies the distribution of ectothermic species and can lead to population extinctions, contributing to increasing ecosystem vulnerability [11], [13]–[15]. In pristine or least impaired ecosystems, temperature variations (e.g. nychtemeral or seasonal) have major driving effects not only on organism physiology, population or community composition, structure and dynamics but also on ecosystem functioning. In the global warming context, an increase of about 0.2°C per decade in the mean global temperature has been expected for the next two decades and numerous models have predicted a 1.8 to 4°C increase by the year 2100 [9]. In addition, the increase in mean temperature would be associated with increasing temperature fluctuations and more frequent extreme events [16], which may lead to more frequent and severe physiological stresses for organisms. In this context, a major concern is to consider and investigate temperature effects on organisms to increase the relevance of ecological and ecotoxicological risk assessment approaches.

Freshwater amphipods (crustaceans) are ectotherms that have been largely used as test organisms in ecotoxicological studies on trace metals, especially the genus *Gammarus* [17]–[19], because of their wide distribution, high abundance [20], clear sexual dimorphism, ease to be collected, sensitivity to various toxicants [21], [22] and trophic importance in freshwater ecosystems. As shredder, *Gammarus* plays a key functional role in leaf litter breakdown processes and, consequently, in freshwater food chains and nutrient cycling [23]–[25]. Moreover, the accumulation of pollutants in *Gammarus* has been recognized as a relevant tool for water quality and environmental risk assessment (e.g. in monitoring or routine tests) via the assessment of contamination level, uptake and elimination rates and bioconcentration factors [20].

Gills which represent the major site of penetration and concentration of metals in many freshwater organisms [26] are crucial for oxygen uptake, acid–base balance, osmotic and ionic regulation [27]–[28]. Crustaceans exposed to pollutants, environmental stressors and/or pathological agents usually exhibit a disruption of the iono/osmoregulation mechanisms, including alterations in the structure and ultrastructure of the branchial and excretory organs, and changes in the Na^+^/K^+^-ATPase activity, ion fluxes and gill surface permeability [29]. As a result, Na^+^ and Cl^−^ which are known to make up to 90% or more of the haemolymph osmotic pressure in most crustaceans [30], can be impaired by chemical stress (e.g. zinc [31]; copper [32]; cadmium [17]; acidification [33]). Osmoregulation is an important and highly energy-consuming regulatory function in aquatic invertebrates. According to Sutcliffe (1984, [34]), 11% of the total energy budget in *G. pulex* is devoted to osmoregulation. In the past few years, several studies have used behavioural responses as tools for ecotoxicity testing and water quality monitoring (e.g. locomotor activity [35]–[36]; ventilatory activity [37]–[38]; feeding rate [39]). The development of such behavioural tests is of strong interest for ecotoxicology because, in addition to being sensitive, fast, simple to perform and cheap, they allow to link the toxic effects obtained at biochemical/cellular levels to the impacts observed on populations and communities [35]. The organism mobility is an ecologically relevant behavioural marker as well, since locomotion is required to find food, escape from predators and obtain mating success. Any pollutant that interferes with mobility is likely to reduce the fitness of organisms and could involve ‘ecological death’ [40].

We hypothesized that (i) metals alone or in mixture would be concentrated by gammarids; and (ii) adverse effects on osmoregulation would lead to alteration in behaviour (i.e., locomotor activity), since energetic allocation should enhance maintenance functions (such as osmoregulation and detoxification).

The objectives of the present study were to 1) investigate physiological and behavioural responses of *G. pulex* exposed to Cd and AsV alone, 2) compare responses due to individual metal stress to those obtained under binary metal exposure, and 3) better assess and understand effects and action mechanisms of these two metals alone and in interaction under different temperatures (5, 10 and 15°C). To match these goals, different levels of Cd and AsV, alone or in binary mixture, were used to assess their effects on survival, bioconcentration (i.e. [AsV] or [Cd] in body tissues), behavioural (ventilatory and locomotor activities) and physiological responses (iono-regulation of [Na^+^] and [Cl^−^] in haemolymph). The interactive effects of binary mixtures were evaluated for each tested temperature using a predictive model [41]–[42].

## Methods

### Ethics Statement

All necessary permits were obtained for the described field studies. We obtained the authorization to sample on our field study site by the Meuse prefecture, the regional representative of the French state. The field study did not involve endangered or protected species.

In France, working with *Gammarus* does not require permission, *Gammarus pulex* is not a protected species and its use in scientific researches does not require any specific permit. All efforts were made to reduce the suffering of animals. Non used individuals (females and excess of males) in laboratory experiments were rapidly returned to the collection site.

### Collection and Acclimation of Organisms

Adult males of *G. pulex,* used as test organisms in this study, were collected in winter with both a hand net and artificial substrates from an unpolluted stream, the Méholle River [vicinity of Void-Vacon, FRANCE, Lambert II E (X): 841244; Lambert II E (Y): 2412346]. Amphipods were quickly brought to the laboratory in plastic vessels. Specimens were sexed using sexual dimorphism characters, with males exhibiting more massive gnathopodes than females [43]–[44]. Males were measured from the top of the cephalothorax to the base of the telson using image software analysis (Studio version 7; ImageJ software, Version 1.42q). Mean body size was 11.0±1.1 mm. Adult males were acclimatized in oxygenated aquaria (approximately 300 males/aquarium) during a week in 5 L of mineral water (Volvic, France) under constant temperature and photoperiod conditions (5.0, 10.0 or 15.0±0.6°C; 16 h of light/8 h of darkness) before being used in experiments. Specimens were fed with discs of conditioned leaves during acclimation up to 24 h before experiment. During acclimation, a daily renewal of both Volvic water and food was performed.

### Experimental Design

**Figure 1:**
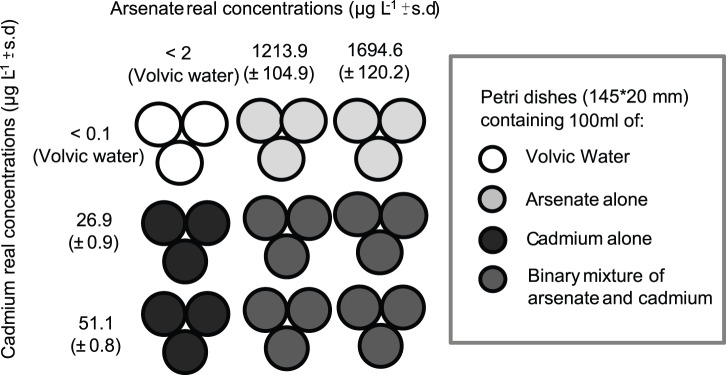
Experimental design and mean real concentrations of arsenate and/or cadmium tested during the experiments. Values (µg L^−1^± s.d.) were measured by graphite furnace atomic - absorption spectrophotometry (AAS Varian SpectrAA-330, detection limits: 2 µg_As_ L^−1^ and 0.1 µg_Cd_ L^−1^) or by flame atomic – absorption spectrophotometry (AAS PERKIN ELMER AAnalyst 100, detection limits: 20 µg_Cd_ L^−1^) Mean real concentrations were based on n = 12 for control and n = 36 for all the combined “temperature x tested conditions”.

After the acclimation period, groups of twenty-three males of *G. pulex* were transferred from batches to plastic Petri dishes (145 mm width and 20 mm height;  =  ‘dishes’ hereafter), each containing 100 mL of tested toxic solution (experimental dishes) or Volvic natural mineral water (control dishes; [Ca^2+^] = 11.5 mg L^−1^; [Mg^2+^] = 8 mg L^−1^; [Na^+^] = 11.6 mg L^−1^; [K^+^] = 6.2 mg L^−1^;[SO_4_^2−^] = 8.1 mg L^−1^; [HCO_3_^−^] = 71 mg L^−1^; [NO_3_^−^] = 6.3 mg L^−1^; [Cl^−^] = 13.5 mg L^−1^; [SiO_2_] = 31.7 mg L^−1^; [F^−^] = 0.22 mg L^−1^; pH = 7.0) and nine glass pebbles (Ø 15 mm, convex side down). As gammarids were not fed during the whole experiment, glass pebbles served both as substrate and refuge for animals to avoid at best cannibalism. For each acclimation temperature, three replicates of twenty-three gammarids were exposed during 96 hours to (i) two concentrations of AsV alone, (ii) two concentrations of Cd alone and (iii) all the possible binary combinations of AsV and Cd with the previous concentrations (Figure 1). In order to avoid chemical variations (in metal concentrations) during the 96-hour exposure, the water was renewed every 24 h after the daily survival count. pH was measured every day and remained stable in the tested solutions (7.27±0.15) independently of their Cd and/or AsV concentrations.

Test containers and dishes were filled up with metal solutions one week before the beginning of experiments, allowing a chemical equilibrium before experiment onset, thus minimizing metal adsorption during experiments. Chemicals used were provided by SIGMA-Aldrich, UK. Stock solutions of the metals (10 mg_Cd_ L^−1^ and 100 mg_AsV_ L^−1^) were prepared by dissolving cadmium chloride (CdCl_2_) and sodium arsenate heptahydrate (AsHNa_2_O_4;_ 7H_2_O) in milliQ water and then were stabilized with 0.1% of HNO_3_ (65%, Suprapure, Merck). Metal test solutions were prepared using mineral water (Volvic, France) as diluent of stock solutions. Using the Excel macro REGTOX [45] on preliminary results of dose-effect experiments allowed to independently identify the [AsV] and [Cd] causing 10 and 25% mortality of *G. pulex* (i.e. LC10 and LC25, respectively) in 96 h (see Table 1) at 10°C. Mortality was processed using a logistic curve-fitting procedure applying the method described by Vindimian et al. [46] and Isnard et al. [47]. Corresponding results were used to build up a complete experimental design of binary mixtures, in which selected AsV and Cd concentrations were chosen close to these lethal concentrations (Figure 1). Three different experimental concentrations for both Cd and AsV were prepared from the acclimation water (Volvic water) using 10 mg_Cd_ L^−1^ and 100 mg_AsV_ L^−1^ of stock solutions (nominal concentration: control = 0 µg_AsV_, As1 = 1220 µg_AsV_, As2 = 1700 µg_AsV_ L^−1^ and control = 0 µg_Cd_ L^−1^, Cd1 = 27 µg_Cd_ L^−1^, Cd2 = 52 µg_Cd_ L^−1^; real concentrations, mean±S.D.: control <5 µg_AsV_ L^−1^, As1 = 1213.9±104.9 µg_AsV_ L^−1^, As2 = 1694.6±120.2 µg_AsV_ L^−1^ and control <0.1 µg_Cd_ L^−1^, Cd1 = 26.9±0.9 µg_Cd_ L^−1^, Cd2 = 51.1±0.8 µg_Cd_ L^−1^).

**Table 1:** Mean LC10 and LC25 values (µg L^−1^±95% confidence intervals) estimated for *Gammarus pulex* after 96-hour of arsenate or cadmium exposure using REGTOX Excel macro (Vindimian et al., 1983) based on Hill model.

	LC10 (µg L^−1^)	LC25 (µg L^−1^)
Arsenate	1121.2 (893.5–1635.5)	1645.0 (1334.5–2110.2)
cadmium	28.2 (13.9–30.3)	54.1 (45.0–69.9)

### Mortality Rate and Evaluation of the Interactive Mode of Action

Every 24 h, dead animals were removed from each dish and living ones were counted in the three replicates of 23 animals. The theoretically expected mortality of *G. pulex* subjected to the interactive toxic effects of binary mixtures was derived from a simple mathematical model. This model, based on the theory of probabilities, assumes that the chemicals are acting (antagonistically, additively or synergistically) simultaneously on the affected organisms [42], [48]–[50]. In this model, if M1 is the mortality (i.e. “inhibition rate” *sensu* [42], [49]) caused by a given concentration of chemical A1 and M2 the mortality caused by a given concentration of chemical A2, then the theoretically expected mixture toxicity ME, when the two chemicals are applied together, is given by the following equation:
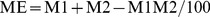
(1)For each binary mixture, the experimentally observed mortality in 96 h (MO) was statistically compared to the theoretically expected mixture mortality ME calculated with equation (1) using a Student t-test (n = 5), after verification of the normal probability distribution of the observed values. If MO and ME were not significantly different (p≥0.05), then the mode of interaction was characterized as additive. If MO was significantly lower than ME, the mode of interaction was considered as antagonistic. If MO was significantly higher than ME, the mode of interaction was considered as synergistic.

### *Gammarus* [AsV] and [Cd]

Metal concentrations in body tissues were measured after 96-hour exposure of *G. pulex* to various treatments. Three replicates of at least five alive gammarids were composed per treatment. Gammarids were rinsed with nanopure water and dried on filter paper. They were immediately stored at −20°C in zipped bags. Then, gammarids were dried in a drying kiln at 65°C for 48 h before dry weight measurement. Biological material was digested for 48 h at 65°C with 1 mL HNO_3_ diluted in half (65%, suprapure, MERCK). Final samples and standard solutions were adjusted to concentration of 2% HNO_3_ by addition of 4 mL of Nanopure water. Metal quantification in biological samples was performed using graphite furnace for AsV (arsenic total) or flame-atomic absorption spectrophotometry for Cd (AAS). All metal concentrations in biological tissues have been reported in µg metal g dry weight^−1^.

### Analysis of Haemolymphatic [Na^+^] and [Cl^−^]

Samples of haemolymph (0.8–1.2 µL) were transferred to a gauged 5-µl microcapillary tube and were centrifuged for 10 min at 6596 *g*. After centrifugation the liquid phase was diluted in 2 mL of Nanopure water. Sodium concentrations in haemolymph were evaluated using atomic absorption spectrophotometry (Perkin Elmer Analyst 100) whereas chlorides were measured by ionic chromatography (Dionex 4500i equipped with an Ion Pac AS4A column). Further details on haemolymph sampling and analysis are available in [33].

### Ventilatory Activity

The pleopods are specialized abdominal appendages in malacostraceans used both for swimming and for facilitating respiratory gas exchanges across tegument. The ventilatory activity was recorded by the observation of the pleopod beat frequency during 1 min [51]. The measurements were performed rapidly at the same period of the day (fixed hours: 13 h–14 h) to avoid the effects of a possible circadian rhythm of respiration [52].

### Locomotor Activity

Locomotor activity was monitored by counting the number of moving animals in 800 mL tank containing 10 individuals [53]. A piece of net was added in the tank to provide a resting surface (mesh size 200 µm, length×width: 6×5 cm). Each count of moving *G. pulex* was conducted on a period of 2 seconds and was repeated 35 times. Measurements were performed at the same period of the day, with similar light conditions and without noise.

### Statistical Analyses

The comparison of observed physiological and behavioural responses between tested conditions was performed using two-way ANOVA followed, if a significant heterogeneity (i.e. p<0.05) had been identified, by post hoc LSD Fisher’s tests. For responses expressed in % (mortality and locomotion), an arcsin root square transformation was done prior to applying statitistical tests. The weighted means were calculated with Statistica software to identify (i) the “condition” effects (control *vs.* metal exposures) when all temperatures were combined and (ii) the “temperature” effect when the results from all metal conditions were combined.

## Results

### Mortality Rate

**Figure 2:**
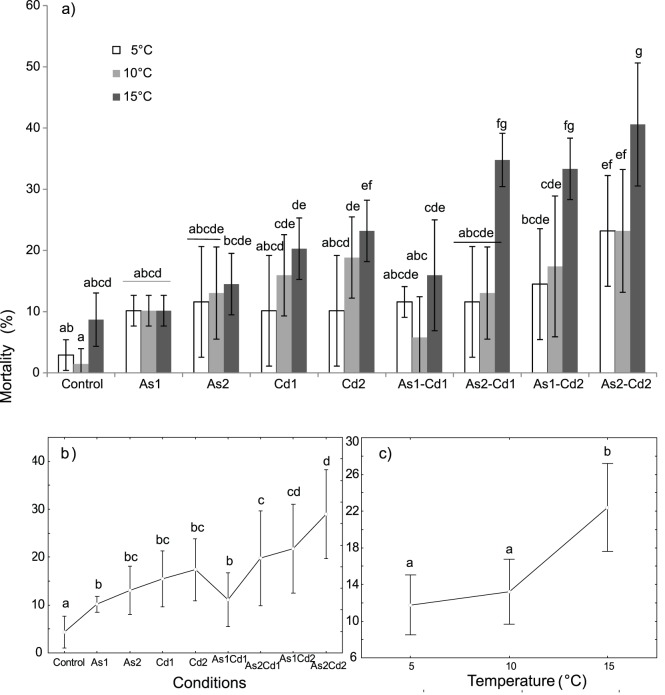
Mean mortality (%) in *Gammarus pulex* exposed to different conditions at three temperatures (5-10-15°C). The different conditions were “control”, “arsenate”, “cadmium” and four binary mixtures. ***a) Temperature × condition effect:*** Mean mortality for each tested condition and temperature. Vertical bars represent standard deviations. ***b) Condition effect:*** Weighted mean mortality for each tested condition (results for all the tested temperatures were combined). Vertical bars represent 0.95 confidence intervals. ***c) Temperature effect:*** Weighted mean mortality for each temperature (results for all the tested conditions were combined). Vertical bars represent 0.95 confidence intervals. Letters (a to g) were used as labels to illustrate significant differences in mean mortality values (two-way ANOVAs + LSD Fisher post hoc tests; at p<0.05 level of significance).

Similar *G. pulex* mortality rates were reported in controls, the highest one was observed at 15°C (2.9±2.5 to 8.7±4.3%, Figure 2a). Significant effects of both temperature and exposure conditions were observed on mortality rate (Table 2; two-way ANOVA, p<0.05) without any significant effect of the interaction (temperature x conditions).

**Table 2:** Results of the two-way ANOVAs (*p<0.05; ** p<0.01; ***p<0,001) testing the homogeneity of the physiological and behavioural responses of *Gammarus pulex* after 96 h exposure at 5, 10 and 15°C (temperature effect) to different concentrations of arsenate; cadmium and binary mixtures of these two metals (condition effect).

	Mortality	Concentrations in body tissues		Locomotion	Ventilation	[Na^+^]	[Cl^−^]
		AsV	Cd				
T°C	1.10^–6^ ***	0.11	2.6.10^–4^ **	<10^–6^***	<10^–6^***	5.5.10^–3^**	1.5.10^–5^***
Conditions	<10^–6^***	<10^–6^***	<10^–6^***	<10^–6^***	1.9.10^–3^**	8.6.10^–3^**	1.1.10^–4^***
T°C*conditions	0.18	0.10	3.7.10^–2^*	<10^–6^***	3.4.10^–4^***	1.5.10^–3^**	1.10^–6^***

When all the conditions were considered together, the rise in temperature tended to increase the mortality rate of *G. pulex* (Figure 2c). At 10°C, the mortality rate was close to that observed at 5°C (13.2±6.8% and 11.7±6.8%, respectively) but significant differences were observed between 15°C (22.4±5.6%) and the previous temperatures. The mortality rate increased significantly (i) between 5°C and 15°C for individuals exposed to Cd2 alone, and (ii) between 15°C and both 5°C and 10°C for individuals exposed to the binary mixtures As1Cd2; As2Cd1 and As2Cd2 (Fig. 2a).

In both single exposures of AsV or Cd, mortality rates were concentration-dependent and increased with the rise of metal concentration (Fig. 2a, 2b). Significant differences in mean mortality rates were found between control and exposed individuals when combining the results from all the tested temperatures (Fig. 2b). However, for AsV alone, no significant differences in mortality were observed between control, As1 and As2 for each tested temperature (Figure 2a). In contrast, for single Cd exposure, significant increases in mortality were found between control and exposed individuals at 10°C (control *vs.* Cd1 or Cd2) and 15°C (control *vs.* Cd2; Fig. 2a). However, no significant increase in mean mortality was observed between Cd1 and Cd2 when all the temperatures were combined (Fig. 2b).

In binary mixtures containing the same Cd concentration, an increase in AsV concentration tended to increase the mortality rate (e.g. As1Cd1 *vs.* As2Cd1; As1Cd2 *vs.* As2Cd2; Fig. 2b), but a significant difference was only observed at 15°C for As1Cd1 *vs.* As2Cd1 (Fig. 2a). Similarly, the average mortality rates slightly increased along the gradient of Cd in binary mixtures containing the same concentration of AsV (i.e. As1 or As2) but the only one significant difference was observed for As1 (As1Cd1 *vs.* As1Cd2) at 15°C (Fig. 2a).

**Figure 3:**
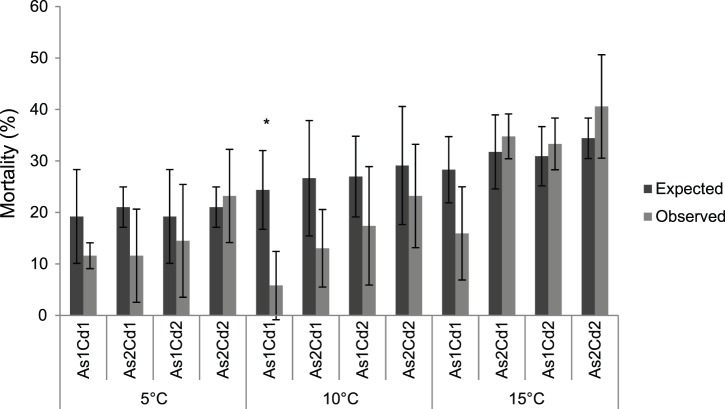
Comparison of mean mortality (± s.d.) between ‘theoretically expected’ and ‘observed’ combined effects. Mean mortality obtained for different cadmium/arsenate binary mixtures in *Gammarus pulex* after 96-hour exposure. An asterisk indicated a significant difference between the observed and expected values for a given binary mixture (Student-t test, p<0.05).

Globally, both the predicted and observed mean mortalities of *G. pulex* in the different binary mixtures increased with the rise in temperature but also along the gradient of AsV and Cd concentrations in mixtures (Fig. 2a, c and Fig. 3). For all the binary mixtures, the predicted mean mortality (minimal-maximal mean values) after 96 h exposure varied between 19.2–21.0% at 5°C; 26.6–29.1% at 10°C; 28.3–34.4% at 15°C while the observed mean mortality varied between 11.6–23.2% at 5°C; 5.8–23.2% at 10°C and 15.9–40.6% at 15°C (Fig. 3). These results suggested a lower toxic effect of mixtures of Cd and AsV on the freshwater amphipod *G. pulex* at 5 and 10°C, than theoretically expected. In contrast, when gammarids were exposed to mixtures of Cd and AsV at 15°C, the observed toxic effect was slightly higher than theoretically expected (excepted in the As1Cd1 mixture). Nevertheless, the differences between observed and expected mortalities were not significant (t-test; p>0.05) for all the conditions (except As1Cd1 at 10°C, Fig. 3). Then, assuming the hypothesis that both metals were simultaneously acting, the interaction between AsV and Cd led to an ‘additive’ toxicity for all the tested binary mixtures, excepted for As1Cd1 at 10°C where AsV and Cd had an ‘antagonistic’ effect on toxicity.

### [AsV] in Body Tissues

**Figure 4:**
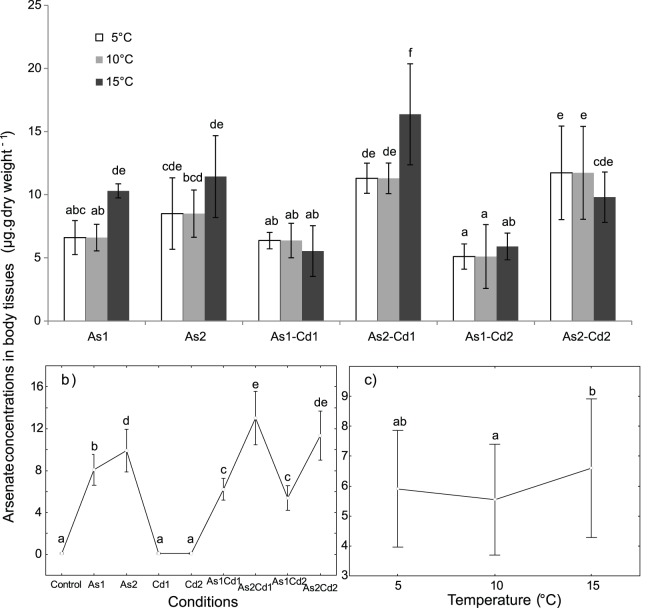
Mean arsenate concentrations in *G. pulex* body tissues (µg. g dry weight^−1^). Concentrations obtained for individuals exposed 96 h to different conditions (control, arsenate, cadmium and four binary mixtures) at three temperatures (5-10-15°C). ***a) Temperature × condition effect:*** Mean internal arsenate concentration in *G. pulex* for each tested condition and temperature. Vertical bars represent standard deviations. ***b) Condition effect:*** Weighted mean concentration of internal arsenate for each tested condition (results for all the tested temperatures were combined). Vertical bars represent 0.95 confidence intervals. ***c) Temperature effect:*** Weighted mean concentration of internal arsenate concentrations for each temperature (results for all the tested conditions were combined). Vertical bars represent 0.95 confidence intervals. Letters (a to f) were used as labels to illustrate significant differences in mean [AsV] values (two-way ANOVAs + LSD Fisher post hoc tests; at p<0.05 level of significance).

In the controls, for all the tested temperatures, the [AsV] in body tissues was lower than the relative detection limit when measured by graphite furnace absorption spectrophotometry (2 µg_As_ L^−1^). A significant effect of ‘condition’ on the [AsV] in body tissues was observed (Table 2, two-way ANOVA, p<0.05), but the effects of ‘temperature’ or interaction [temperature × condition] were not significant. The rise in temperature did not clearly influence the [AsV] in the body tissues of *G. pulex* (Fig. 4a, c) even if the [AsV] in body tissues was higher at 15°C than at 5 or 10°C for As1 and As2Cd1 (p<0.05; Fig. 4a).

In single AsV exposure, the [AsV] in body tissues was slightly higher in As2 than in As1 exposure but the observed differences were never significant for all the tested temperatures (Fig. 4a). However, when gathering the three temperatures, the average body tissue concentration significantly increased with external [AsV] (Fig. 4b).

In the binary mixtures containing the same [Cd], the [AsV] in body tissues increased significantly along the gradient of AsV (As1Cd1 *vs.* As2Cd1; As1Cd2 *vs.* As2Cd2) when the tested temperatures were considered both separately or together (Fig. 4a,b). In the binary mixtures containing As1, the [AsV] in body tissues varied in the same range along the gradient of Cd whatever the tested temperature (As1Cd1 *vs.* As1Cd2). Moreover, the addition of Cd with As1 has engendered a decline in internal [AsV] when compared to As1 alone (As1 *vs.* As1Cd1, As1 *vs.* As1Cd2), but the difference was statistically significant only at 15°C (Fig. 4a). For the binary mixtures containing As2, the [AsV] in body tissues varied in the same range along the gradient of Cd concentration in mixtures (As2Cd1 *vs.* As2Cd2) at 5 and 10°C. However, a significant decrease in internal [AsV] was observed at 15°C. When compared to As2 alone, the addition of Cd1 increased the internal [AsV] in exposed individuals (As2Cd1), while the addition of Cd2 (As2Cd2) did not changed the internal [AsV].

### [Cd] in Body Tissues

**Figure 5:**
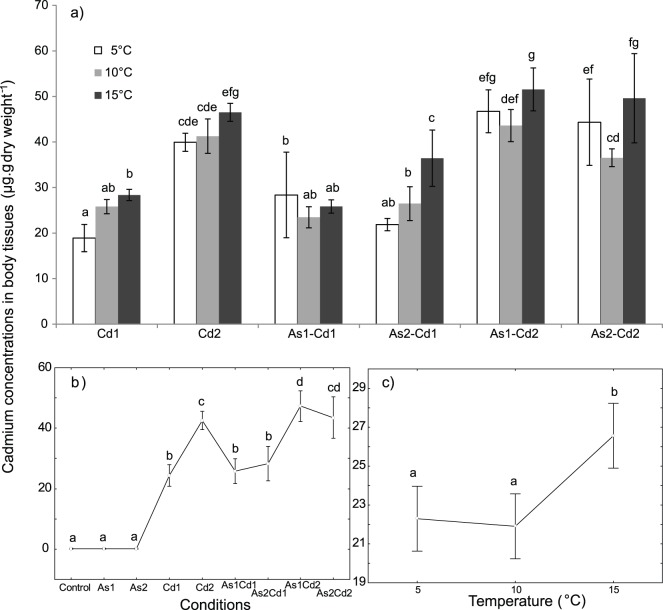
Mean cadmium concentrations in *G. pulex* body tissues (µg. g dry weight^−1^). Concentrations obtained for individuals exposed 96 h to different conditions (control, arsenate, cadmium and four binary mixtures) at three temperatures (5-10-15°C). ***a) Temperature × condition effect*** *.* Mean internal Cd concentrations *in G. pulex* for each tested condition and temperature. Vertical bars represent standard deviations. ***b) Condition effect:*** Weighted mean concentration of internal Cd for each tested condition (results for all the tested temperatures were combined). Vertical bars represent 0.95 confidence intervals. ***c) Temperature effect:*** Weighted mean concentration of internal Cd for each temperature (results for all the tested conditions were combined). Vertical bars represent 0.95 confidence intervals. Letters (a to g) were used as labels to illustrate significant differences in mean [Cd] values (two-way ANOVAs + LSD Fisher post hoc tests; at p<0.05 level of significance).

In the control conditions, for all the tested temperatures, the [Cd] in body tissues was lower than their relative detection limits measured by graphite furnace or atomic-absorption spectrophotometry (0.1 µg _Cd_ L^−1^). Significant effects of (i) temperature, (ii) condition and (iii) interaction [temperature x condition] on the [Cd] in body tissues were observed (Table 2, two-way ANOVA, p<0.05). The average [Cd] in body tissues of *G. pulex* increased between 5 or 10°C and 15°C when all the exposure conditions were considered together (Fig. 5c). An interaction [temperature x condition] was revealed and the internal [Cd] was significantly higher (i) at 15°C when compared to 5°C for individuals exposed to Cd1 or As2Cd1, and (ii) at15°C when compared to 10°C for individuals exposed to As1Cd2 or As2Cd2 mixtures (Fig. 5a).

In single Cd exposure, the [Cd] in body tissues was significantly higher for the external [Cd2] whatever the tested temperature (Fig. 5a, b). When the tested temperatures were considered together, the internal [Cd] was higher in the binary mixtures containing Cd2 than in the binary mixtures containing Cd1 (Fig. 5b). Moreover, an addition of AsV in the binary mixtures containing Cd1 or Cd2 did not significantly changed the internal [Cd] when compared to the internal [Cd] for Cd1 or Cd2 exposure. However, when considering separately the different temperatures, the relationship between the internal [Cd] in body tissues and the external [Cd] in binary mixtures was less clear, with a significantly higher [Cd] in body tissues (i) at 5°C for As1Cd1 (if compared to Cd1) and (ii) at 15°C for As2Cd1 (if compared to Cd1; Fig. 5a).

### Locomotion

**Figure 6:**
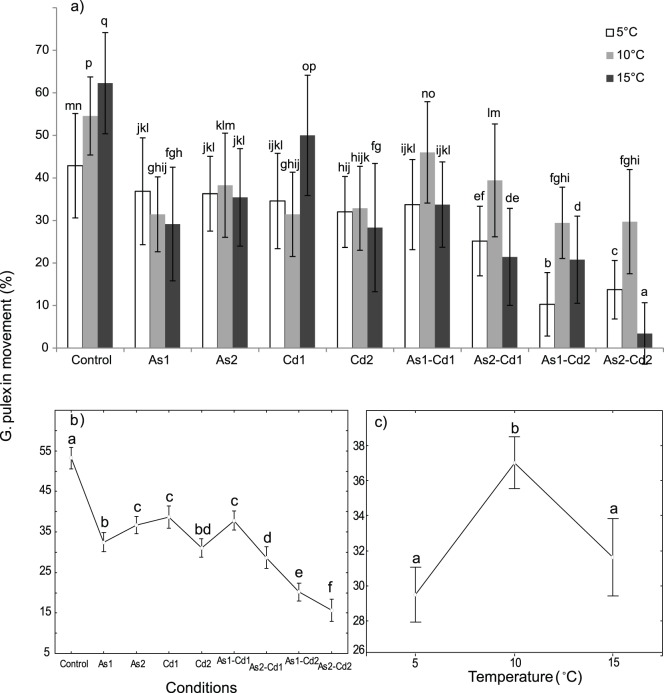
Mean mobility of *Gammarus pulex* (in %) exposed to different conditions. Mobility obtained for individuals exposed 96 h to different conditions (control, arsenate, cadmium and four binary mixtures) at three temperatures (5-10-15°C). ***a) Temperature × condition effect:*** Mean mobility (%) for each tested condition and temperature. Vertical bars represent standard deviations. ***b) Condition effect:*** Weighted mean locomotor activity for each tested condition (results for all the tested temperatures were combined). Vertical bars represent 0.95 confidence intervals. ***c) Temperature effect:*** Weighted mean locomotor activity for each temperature (results for all the tested conditions were combined). Vertical bars represent 0.95 confidence intervals. Letters (a to q) were used as labels to illustrate significant differences in mean mobility values (two-way ANOVAs + LSD Fisher post hoc tests; at p<0.05 level of significance).

In the control conditions, the mobility of *G. pulex* significantly increased with temperature (42.9±12.3% at 5°C; 54.6±9.2% at 10°C and 62.3±11.9% at 15°C; Fig. 6a). Highly significant effects of (i) temperature, (ii) condition and (iii) interaction [temperature x condition] were observed on the locomotion of *G. pulex* (Table 2, two-way ANOVA, p<0.05).

When all the exposure conditions were considered together, gammarids were significantly more mobile at 10°C than at the two other temperatures, for which no differences were reported (37.0±10.6% *vs.* 29.5±9.6% at 5°C and 39.6±11.6% at 15°C; Fig. 6c). Considering the results independently obtained for each tested temperature (Fig. 6a), the higher locomotor activity of gammarids at 10°C was mainly due to its responses in binary mixtures. The organisms exposed to metal were significantly less mobile than those non-exposed, when the temperatures were combined (Fig. 6b). When the individuals were exposed to single AsV concentrations, their mobility rate decreased with increasing temperature for As1, but no significant difference was observed for As2 (Fig. 6a),The mobility of the individuals exposed to single Cd concentrations increased between 5 or 10°C and 15°C for Cd1, while it slightly decreased for Cd2 (Fig. 6a).

In the two single AsV exposures, the mobility of *G. pulex* was slightly higher in As2 than in As1 (36.7±1.8% *vs.* 32.5±2.4%; Fig. 6b), when the tested temperatures were considered separately. These differences were significant at 10°C and 15°C but not at 5°C (Fig. 6a). In the two single Cd exposures, the mobility of *G.* *pulex* varied in the same range (32%), except at 15°C where the mobility was higher with Cd1 (50%) than with Cd2 (28%; Fig. 6a). This difference was strong enough to produce a significant decrease in the *Gammarus* mobility between Cd2 and Cd1 when all the tested temperatures were considered together (Fig. 6b). In these conditions, the mobility of *Gammarus* decreased also in the binary mixtures containing Cd2, when compared to those containing Cd1 (Fig. 6c). The same trend was found in the mixtures containing As2, when compared to those containing As1. Locomotion was systematically lower in the binary mixtures containing Cd2 than in the binary mixtures containing Cd1. An addition of AsV in the binary mixtures containing Cd1 or Cd2 decreased the mobility of *G. pulex* when compared to its mobility in single Cd1or Cd2 exposure. An addition of Cd in As1 or As2 tended also to decrease the mobility of *G. pulex* when compared to that in the corresponding single AsV exposure (Fig. 6b): Significant differences were observed between As2Cd1 and Cd1 or As2, As1Cd2 and Cd2 or As1, As2Cd2 and Cd2 or As2.

When the temperatures are considered separately, the individuals exposed at 5°C and 15°C in binary mixtures mainly exhibited significantly lower mobility that the individuals impaired by the corresponding concentrations of Cd or AsV alone, while at 10°C, higher or similar mobility was mainly observed (Fig. 6a).

This pattern probably explains why no significant mobility decrease was observed between As1Cd1 and Cd1 or As1 when the temperatures were considered together. Mobility decreased with an increase in Cd mixture. The same significant pattern was observed for AsV mixed with Cd1, but the only significant difference in AsV mixed with Cd2 was observed at 15°C.

### Ventilation

**Figure 7:**
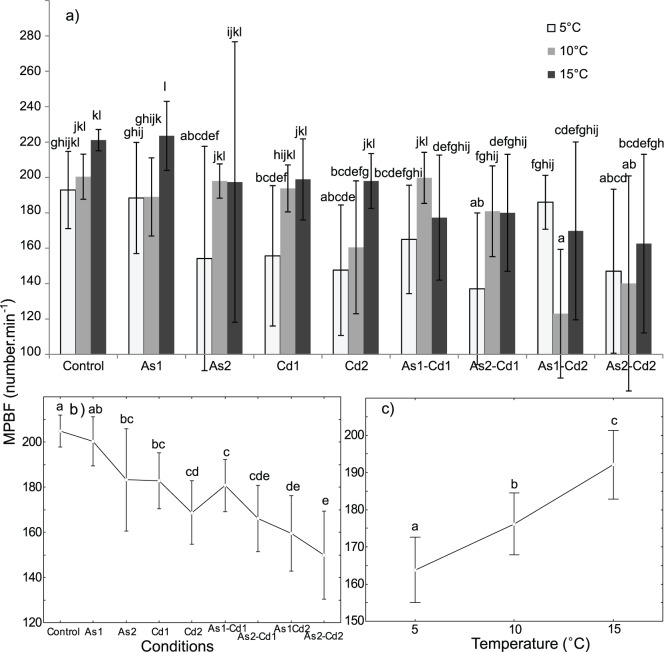
Mean pleopod beat frequency (MPBF) of *Gammarus pulex.* PBF obtained for individuals exposed 96 h to different conditions (control, arsenate, cadmium and four binary mixtures) at three temperatures (5-10-15°C). a) ***Temperature × condition effect:*** Mean MPBF for each tested condition and temperature. Vertical bars represent standard deviations. b) ***Condition effect:*** Weighted mean MPBF for each tested condition (results for all the tested temperatures were combined). Vertical bars represent 0.95 confidence intervals. c) ***Temperature effect:*** Weighted mean MPBF for each temperature (results for all the tested conditions were combined). Vertical bars represent 0.95 confidence intervals. Letters (a to l) were used as labels to illustrate significant differences in MPBF values (two-ways ANOVA + LSD Fisher post hoc tests; at p<0.05 level of significance).

In the control conditions, the ventilation of *G. pulex* slightly increased from 5 to 15°C (i.e. 192.9±21.8 to 221.1±6.0 pleopod beat min^−1^, Fig. 7a), but these differences were not statistically significant. Significant effects of (i) temperature, (ii) condition and (iii) interaction [temperature x condition] on *G. pulex* ventilation were observed (mean pleopod beat frequency - ‘MPBF’ hereafter- table 2, two-way ANOVA, p<0.05).

When all the exposure conditions were simultaneously considered, a significant MPBF increase was observed with rising temperature (163.7±14.1; 176.2±16.6 and 192.1±22.5 pleopod beat min^−1^ at 5, 10 and 15°C respectively; Fig. 7c). When the conditions were considered separately (Fig. 7a), the response pattern was more complex even if the same trend was observed in the control conditions and in the single AsV or Cd exposures (but not in mixtures).

When all the temperatures were simultaneously considered, the individuals exposed to metal (except As1) showed significantly lower ventilation rate than those non-exposed (Fig. 7b). Except at 10°C for AsV, a significant MPBF decrease was observed with an increase in AsV or Cd concentration in the single exposure conditions (Fig. 7a, b). For the tested concentrations, MPBF seemed to be lower for gammarids exposed to Cd than for those exposed to AsV (Cd1 *vs.* As1; Cd2 *vs.* As2) but the difference was not significant.

For the binary mixtures, temperature had a less clear effect on ventilation with either an increase or a decrease in MPBF with rising temperature (Fig. 7a). However, when all the temperatures were simultaneously considered, MPBF tended to decrease with external metal concentration (Fig. 7b). The gammarids exposed to the binary mixtures As1Cd1 or As2Cd1 and the gammarids exposed to AsV or Cd alone exhibited the same ventilation frequency. When comparing the other mixtures at the single corresponding concentration of metals: (i) a significant increase in MPBF was observed between As1Cd2 and As1 at 5°C and (ii) a significant decrease in MPBF was observed between As1Cd2 and As1 or Cd2 at 10°C and As2Cd2 and As2 or Cd2 at 15°C. MPBF was also lower in the binary mixtures containing Cd2 than in the binary mixtures containing Cd1 (Fig. 7b, significant differences only observed for As1Cd1 and As1Cd2).

### Haemolymphatic [Na^+^] and [Cl^−^]

**Figure 8:**
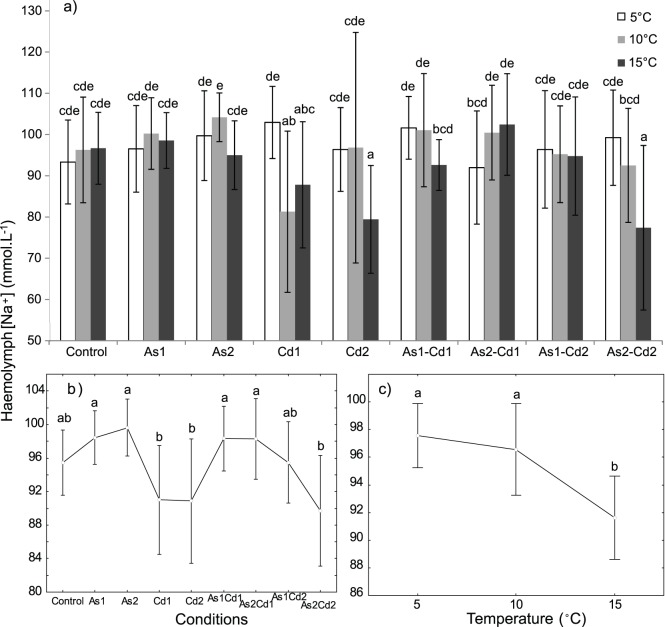
Mean concentrations of haemolymphatic [Na^+^](mmol L^−1^) in *Gammarus pulex.* Concentrations obtained for individuals exposed 96 h to different conditions (control, arsenate, cadmium and four binary mixtures) at three temperatures (5-10-15°C). **a)** ***Temperature × condition effect:*** Mean haemolymphatic [Na^+^] for each tested condition and temperature. Vertical bars represent standard deviations. **b)** ***Condition effect:*** Weighted mean haemolymphatic [Na^+^]for each tested condition (results for all the tested temperatures were combined). Vertical bars represent 0.95 confidence intervals. **c)** ***Temperature effect:*** Weighted mean haemolymphatic [Na^+^] for each temperature (results for all the tested conditions were combined). Vertical bars represent 0.95 confidence intervals. Letters (a to e) were used as labels to illustrate significant differences in values of mean haemolymphatic [Na^+^] (two-way ANOVAs + LSD Fisher post hoc tests; at p<0.05 level of significance).

**Figure 9:**
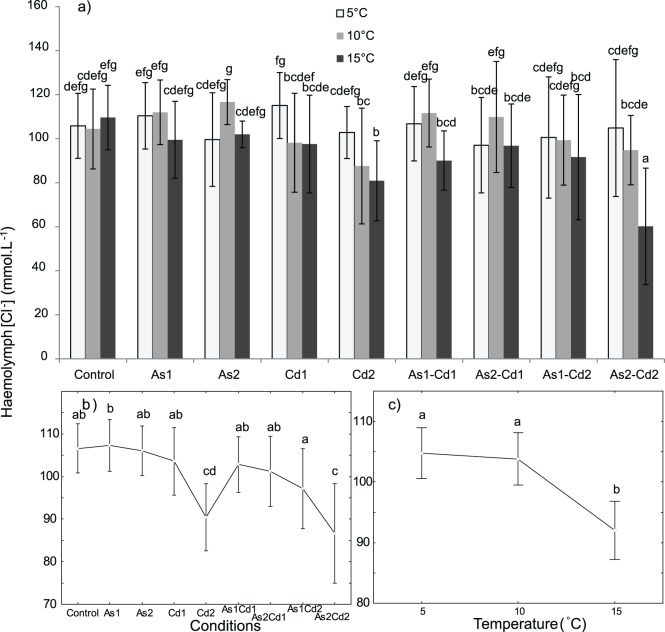
Mean concentrations of haemolymphatic [Cl^−^](mmol L^−1^) in *Gammarus pulex.* Concentrations obtained for individuals exposed 96 h to different conditions (control, arsenate, cadmium and four binary mixtures) at three temperatures (5-10-15°C). **a)** ***Temperature × condition effect:*** Mean haemolymphatic [Cl^−^] for each tested condition and temperature. Vertical bars represent standard deviations. **b)** ***Condition effect:*** Weighted mean haemolymphatic [Cl^−^] for each tested condition (results for all the tested temperatures were combined). Vertical bars represent 0.95 confidence intervals. **c)** ***Temperature effect:*** Weighted mean haemolymphatic [Cl^−^] for each temperature (results for all the tested temperatures were combined). Vertical bars represent 0.95 confidence intervals. Letters (a to g) were used as labels to illustrate significant differences in values of mean haemolymphatic [Cl^−^] (two-way ANOVAs + LSD Fisher post hoc tests; at p<0.05 level of significance).

In the control conditions, the *G. pulex* haemolymphatic [Na^+^] and [Cl^−^] were similar and independent of temperature ([Na^+^]: from 93.3±10.2 to 96.6±8.7 mmol L^−1^, Fig. 8a; [Cl^−^]: from 104.4±18.1 to 109.6±14.7 mmol L^−1^, Fig. 9a), even if the haemolymphatic [Na^+^] and [Cl^−^] slightly increased with rising temperature. We observed highly significant effects of (i) temperature, (ii) condition and (iii) interaction [temperature x condition] on [Na^+^] and [Cl^−^] in the haemolymph of *G. pulex* (Table 2, two-way ANOVA, p<0.05). When all the exposure conditions were simultaneously considered, a significant decrease in both haemolymphatic [Na^+^] and [Cl^−^] was observed between 5–10°C and 15°C, but no significant difference was observed between 5°C and 10°C (Fig. 8c and 9c). When considering each condition independently, the effect of temperature was less clear on [Cl^−^] than on [Na^+^] with either an increase or a decrease in [Cl^−^] with rising temperature. However at 15°C, the haemolymphatic [Cl^−^] was often lower than at 5 or 10°C (Fig. 9a). Whatever the temperature, no significant difference in the haemolymphatic [Cl^−^] or [Na^+^] was observed when the individuals were exposed to single AsV concentrations (if compared to the control conditions; Fig. 8a,b and 9a,b). When single Cd exposures were compared to the control conditions, a decrease in both haemolymphatic [Cl^−^] or [Na^+^] was observed with Cd1 at 10°C and Cd2 at 15°C for Na^+^, and with Cd2 at 15°C for Cl^−^ (Fig. 8a and 9a). Along the [Cd] gradient, both haemolymphatic [Na^+^] and [Cl^−^] tended to slightly decrease in single exposures, but the differences (except for Na^+^ at 10°C) were not significant.

When all the temperatures were simultaneously considered, both the haemolymphatic [Cl^−^] or [Na^+^] of *G. pulex* tended to decrease in mixtures with an increase of only one of the two metals (Fig. 8b and 9b), especially when [Cd] increased. The Cd exposure led to a higher ionoregulation disturbance than the AsV exposure. For the haemolymphatic [Cl^−^], the difference was only significant between As2Cd2 and the three other mixtures. For Na^+^, the differences were significant between As2Cd2 and As1Cd1 or As2Cd1. Moreover, both haemolymphatic [Na^+^] and [Cl^−^] were lower in binary mixtures containing Cd2 than in binary mixtures containing Cd1 (Fig. 8b and 9b).

The haemolymphatic [Cl^−^] did not vary significantly between As1Cd1 or As1Cd2 and the corresponding single AsV or Cd concentrations (Fig. 9a, b). For As2Cd1, no significant difference was observed with single metal exposure except at 5°C where a loss of [Cl^−^] was observed in comparison to Cd1. For As2Cd2, a significant decrease was observed at 10°C and 15°C in comparison to As2, and at 15°C in comparison to Cd2.

Similar haemolymphatic [Na^+^] were observed in the organisms exposed to As1Cd1 in comparison to As1 or Cd1 alone, except at 10°C where [Na^+^] was higher in As1Cd1 than in Cd1 (Fig. 8a). For the As2Cd1 and As1Cd2 mixtures, no significant difference was observed at 5°C and 10°C in comparison to the corresponding single concentration of AsV or Cd (except for As2Cd1 *vs.* Cd1 at 10°C). However, at 15°C, a significant increase was observed in comparison to the corresponding single Cd concentrations. For As2Cd2, significant differences were only observed at 10°C and 15°C with a decrease in the haemolymphatic [Na^+^] in mixtures when compared to As2 alone.

## Discussion

### Effects of Single AsV or Cd Exposure

The mortality of *G. pulex* globally increased when individuals were exposed to AsV or Cd in comparison to non-exposed individuals. The mortality rate was external concentration dependent and tended to increase with increasing metal concentration. If compared to non-exposed individuals, *G. pulex* individuals exposed to Cd alone exhibited (i) higher mortality rate, (ii) higher [Cd] in body tissues, (iii) lower behavioural activities (locomotion and ventilation), and (iv) lower haemolymphatic [Na^+^] or [Cl^−^] (i.e. ion loss). The differences significantly increased along the Cd contamination gradient but only for bioconcentration, locomotion and haemolymphatic [Cl^−^]. In the same way, gammarids exposed to AsV alone showed (i) higher [AsV] in body tissues and (ii) lower behavioural activities than non-exposed individuals. The differences were also amplified along an increasing gradient of AsV. In contrary to Cd, the haemolymphatic [Cl^−^] and [Na^+^] in *G. pulex* were not impaired by [AsV].

In accordance with [4] and [54], higher toxicant concentrations (here internal [Cd] or [AsV]) in exposed organism generally increase its mortality. When internal metal concentrations increase, individuals must invest energy in maintenance mechanisms that are highly energy-consuming, such as detoxification, toxicant elimination, homeostasis maintenance (iono−/osmo-regulation), or cellular repairing mechanisms [17], [55]. In [56], Thomasson et al. demonstrated that individuals could reallocate a part of the energy, usually used for displacement or ventilation, into maintenance mechanisms; giving potential explanation of the lower ventilation and/or locomotion responses of *G. pulex* exposed to Cd or AsV when compared to non-exposed organisms. The competition between AsV and phosphate can alter phosphorylation reactions within electron transport chain or glucose metabolism [57]–[60]. This mimetic effect could theoretically impede glucose metabolism and disrupt energy production [61]. Thus, Vellinger et al. [19] assumed that gammarids allocate more oxygen to energetic metabolism when subjected to AsV contamination. A hyperventilation of *G. pulex* was observed when individuals were exposed 10 days at the lowest tested AsV concentration (821 µg_AsV_ L^−1^), because this hyperventilation induced energetic benefit probably exceeded its energetic cost. In addition, a hypoventilation was observed at the highest AsV concentration tested (>1000 µg_AsV_ L^−1^), which reduced energy depletion due to active movements, the energetic cost of movements being assumed to be higher than their energetic benefit.

The ventilation response can be separated into two steps depending on the duration and intensity of exposure [62]. First, the organisms hyperventilate to compensate respiration and/or osmoregulation alteration (compensation). Second, the organisms hypoventilate, when they are close to death. In this study, *G. pulex* could reduce both its ventilation and locomotion rates when exposed to Cd or AsV alone because (i) the energetic balance between cost and benefit was negative, and/or (ii) the metal altered gill structure and functioning (i.e. higher metal impact under higher ventilation rate), and/or (iii) organisms had no more energy (exhaustion), and/or (iv) individuals reduced their locomotion to save oxygen consumption and preserve their energy stock. However, the observed decrease in both pleopod beats and locomotion could be simply due to the direct metal toxicity leading to a worse physiological state of the exposed individuals (e.g. cellular and/or molecular damages due to lysosomal membrane disruption or lipoperoxidation). Hence, in the case of AsV, Thomas *et al.* [63] and Vahter [64] demonstrated that the absorbed AsV was readily reduced to arsenite, a more toxic form of arsenic, inducing a worse physiological state.

A decrease in haemolymphatic [Na^+^] and [Cl^−^] was observed only in *G. pulex* exposed to single [Cd] during 96 hours. Although a direct effect of metals cannot be excluded, the reduction in locomotor and ventilatory activities seems to be induced by the redirection of energy for osmoregulation compensation mechanisms, such as Na^+^/K^+^-ATPase activity (increasing number per surface of ionocytes), directly linked to energetic pathways [17].

Several studies have shown that (i) osmoregulation represents a good tool for evaluating the physiological state of crustaceans, and (ii) stress (e.g. water-borne pollutants, pathological agents or environmental stressors) usually leads to disturbance in Na^+^ and Cl^−^ regulation and/or in osmoregulatory capacity [17], [29], [65]. The effects of AsV on iono−/osmo-regulation are little documented while they have been well studied for Cd, even if reported patterns are contrasted. In coherence with our results, losses of Na^+^ and Ca^2+^ were reported in the rainbow trout [66] and in the crab *Eriocheir sinensis* [67] exposed to Cd. In contrast, other studies reported no effects of Cd exposure on haemolymphatic [Na^+^] or [Cl^−^] while hypocalcemia was found (e.g. in *Oreochromis mossambicus* [68] and in *G. pulex* [17]). As suggested by Felten et al. [17], “between authors” differences could be explained by differences in experimental design (e.g. Cd concentration, water exposure and/or exposure time): exposure to high [Cd] (≥125 µg L^−1^), in the tested conditions used in [17] leads to haemolymphatic Na^+^ loss (Felten, unpublished results).

Three non exclusive hypotheses could explain the impact of Cd on iono-osmoregulation processes and the absence of AsV impact. First, different routes of entrance exist between the two metals: gills represent the major site of Cd penetration, while the main way of AsV entrance in *G. pulex* was drinking water (see [19]). Metal-binding ligands in gills can be considered as a secondary entrance route of AsV. As gills represent a specialized organ in water and ion transports, it is likely that Cd disrupts more iono-osmoregulation processes than AsV does. Second, the metal capacity to bind with Na^+^/K^+^-ATPase, i.e. the driving force for active Na^+^ uptake across gill epithelium [28], would be different. Brooks and Mills (2003, [32]) have shown that copper could inhibit this enzyme, by interfering with –SH groups on Na^+^/K^+^-ATPase. As Na^+^ in haemolymph was reduced in presence of Cd (but not with AsV), Cd could probably inhibit the Na^+^/K^+^-ATPase whereas AsV could not. Third, Cd can compete with calcium for the same binding site thanks to a similar radius between Cd^2+^ and Ca^2+^ [69] whereas AsV could not. Even if the haemolymph calcium concentration was not studied in this paper, we suspected that Cd could induce the inhibition of calcium absorption and its transport at intracellular level as already demonstrated [8], [17], [70].

### Effects of Binary Mixture of AsV and Cd

In this study, the mode of action of the tested binary mixtures on the mortality of *G. pulex*, was considered as ‘additive’, except As1Cd1 at 10°C for which an ‘antagonistic’ mode of action of the metals was evidenced. An antagonistic effect was already observed on the mortality rate of *G. pulex* simultaneously exposed to AsV and Cd during 10 days at 10°C, but for all the tested concentration combinations [71]. This antagonistic effect was less marked with the highest tested concentration combination (i.e. 28.5 µg_Cd_ L^−1^ and 1502 µg_AsV_ L^−1^). Such differences could be explained by differences (i) in exposure time (96 h *vs.* 240 h), and/or (ii) in tested concentrations of each metal in binary mixture which were higher in the present study (26.9–51.1 µg_Cd_ L^−1^ *vs.* 1.9–28.5 µg_Cd_ L^−1^ for Cd concentration range; 1214–1695 µg_AsV_ L^−1^ *vs.* 274.7–1502 µg_AsV_ L^−1^ for arsenate concentration range) than in [**71**].

**Figure 10:**
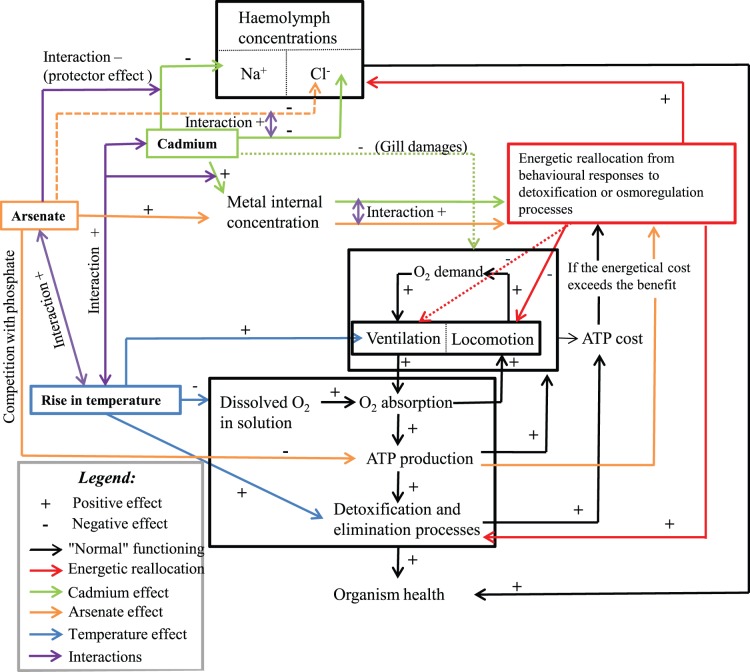
Global physiological model of individual and interactive effects of temperature, arsenate and cadmium in *Gammarus.* A dotted line represents a lower effect than a full one.

In accordance with [62], the relationships between the tested parameters in binary mixtures were less clear than in single metal exposures. For example, in As1Cd2, the [AsV] in body tissues tended to be lower than in single metal exposure while no effect on (i) mortality, and (ii) both locomotion and ventilation were observed when compared to As1 condition (Table 3). This suggests that all these biological parameters have to be considered together to efficiently understand the effects of metal mixtures on the model species (Fig. 10).

**Table 3:** Synthesis of the physiological and behavioural responses of *Gammarus pulex* in binary mixtures in comparison to single corresponding concentrations of arsenate or cadmium (results for all the tested temperatures were combined).

	Mortality		Concentration in body tissues		Locomotion		Ventilation		Na^+^ in haemolymph		Cl^-^ in haemolymph	
	AsV	Cd	AsV	Cd	AsV	Cd	AsV	Cd	AsV	Cd	AsV	Cd
**As1Cd1**	NE	T−	−	NE	+	NE	−	NE	NE	+	T−	T−
**As2Cd1**	T+	T+	+	NE	−	−	−	T−	NE	+	T−	T−
**As1Cd2**	+	T+	−	+	−	−	T−	T−	T−	T+	−	+
**As2Cd2**	+	+	T+	T+	−	−	−	−	−	NE	−	T−

‘NE’: No effect; ‘−’: significant decrease; ‘+’: significant increase; ‘T−’: decrease tendency (but not significant); ‘T+’: increase tendency (but not significant).

In binary mixtures, many authors have shown that one metal could modify the uptake of the other, which is enhanced [72] or inhibited [71], [73], [74]. In other cases, the metal uptake can be unchanged by the presence of the second metal [75]. When combining all the tested temperatures, our results indicated either an increase or a decrease in body tissue metal concentration when compared to single-metal exposures, suggesting either enhancement or inhibition of metal accumulation.

At 10 and 15°C, the presence of Cd2 in As2Cd2 mixture had adverse effects on both haemolymphatic [Na^+^] and [Cl^−^] (Table 3) when compared to As2 effects. Indeed, the haemolymphatic [Cl^−^] decline, not significant in As2Cd1, became significant in As2Cd2. Similarly, no effect of Cd1 was demonstrated on haemolymphatic [Na^+^] while a significant [Na^+^] decline was observed with Cd2. In contrast, the presence of AsV in mixtures with Cd1, seems to have a protector effect on the regulatory capacity of Na^+^, with internal [Na^+^] concentrations close to those observed in the control conditions and higher than those observed in the individuals only exposed to Cd1. However, the “protector effect” of AsV decreased in mixtures with Cd2. Such results could be explained by an increasing alteration of gill structure (and then an increasing mortality) when the external Cd concentration increases in binary mixtures.

The “protector effect” of AsV on Na^+^ ionoregulation could be related to common uptake routes (cf. metal binding proteins in gill surface; [19]). Indeed, if metal binding in gill surface represents a minor way of AsV entrance and a major way of Cd entrance, the two metals could theoretically interact during the branchial uptake. It is likely that AsV would prevent Cd from inhibiting the Na^+^/K^+^-ATPase activity but the mechanism of action remains unknown. The weak AsV “protector effect” on Na^+^ regulation in mixtures containing Cd2 could be explained by (i) higher internal concentration of Cd compared to internal concentration of AsV and (ii) stronger impact of Cd2 on gill structure *in G. pulex*.

AsV and Cd have adverse effects on both locomotion and ventilatory activities in binary mixtures. These effects were more marked for locomotion than for ventilation. Such behavioural modifications could be a strategy for allocating more energy to maintenance functions. However, as the mechanisms of AsV and Cd toxicity (when alone) seem to be different, the organisms exposed to the binary mixture have to detoxify each metal; individuals reducing both locomotion and ventilation activities when compared to individuals subjected to single metal contamination. We hypothesized that the observed behavioural responses could be caused, at least partly, by a higher impact of combined metals on gill integrity.

### Temperature Effects on *G. pulex* Non-exposed to Metals

Mortality rate in *G. pulex* was slightly higher at 15°C than at 5 or 10°C. Numerous studies reported that changes in temperature may affect metabolic rate, locomotion or feeding activities, thus modifying uptake, elimination and detoxication rates [13], [76], [77].

In this study, only changes in behavioural responses were observed; locomotion and ventilatory activities increasing with rising temperature. At 5°C, the metabolism rate was reduced and consequently both locomotion and pleopod beats decreased while osmolality was maintained. Wijnhoven et al. [78] suggested an increasing ventilatory activity of *G. pulex* with rising water temperature because of both higher metabolic rate and depletion in the concentration of dissolved oxygen in the water. Consequently, at 15°C the high *G. pulex* activity requires more dissolved oxygen; leading to an increase in ventilatory activity.

### Temperature Effects on *G. pulex* Exposed to AsV or/and Cd

Along the temperature gradient, the mortality of *G. pulex* only exposed to AsV did not vary significantly whereas the mortality of individuals only exposed to Cd tended to gradually increase. At both 5 and 10°C, the mixtures of Cd and AsV most often induced a lower toxic effect in *G. pulex* than at 15°C. The mode of action of such binary mixtures was considered as ‘additive’, except for As1Cd1 at 10°C which was considered as ‘antagonistic’.

Bat et al. [6] already demonstrated a positive correlation between warming stress and Cd in *G. pulex*. Because the major differences in toxicity were observed for ventilation and physiological parameters between 5 and 15°C, we focused on the interpretation of such differences. The effects of both individual AsV or Cd exposure and thermal stress on *G. pulex* physiological and behavioural parameters may be viewed as a “snowball” effect. Several reviews examining the effects of interactions between temperature and chemicals on aquatic biota [13], [14], [79], [80] reported that an increasing temperature mostly enhances the toxic effect. These authors have also demonstrated that an increasing mortality was often related to an increasing metal uptake and accumulation by the organisms. This higher metal accumulation may be partially explain by changes in speciation and increasing metal bioavailability due to higher solubility of metal compounds, and then higher free ionic metal concentration at elevated temperature [7], [8], [81]. Sokolova and Lannig [10] suggested that metal transport, membrane permeability and systemic functions (e.g. ventilation) can affect temperature–dependent changes in metal uptake and/or elimination. In both single and combined exposures, an increase in temperature enhanced the Cd concentration in body tissues (except for As1Cd1), but no significant temperature impact on AsV concentration in body tissues was demonstrated. Unsurprisingly, the decline in both haemolymphatic [Na^+^] and [Cl^−^] was amplified when individuals were exposed to both (i) single Cd concentration and thermal stress or (ii) metal mixture and thermal stress, if compared to individuals only exposed to Cd (except As2Cd1 *vs.* Cd1). Furthermore, no significant effect on [Na^+^] and only weak effect on [Cl^−^] in haemolymph were observed in *G. pulex* exposed to (i) only AsV and thermal stress, or (ii) metal mixture and thermal stress if compared to individuals only exposed to AsV. In contrast, the ventilatory activity in single or combined metal exposures increased between 5 and 15°C. The ectothermically driven rates of metabolism increase with temperature due to higher energy demand [82]. Consequently, a simultaneous exposure to a given metal (AsV or Cd) and an elevated temperature can increase the oxygen demand of gammarids [83]. In such conditions, *G. pulex* survival needs to achieve enough oxygen to maintain vital functions, e.g. via hyperventilation, even if high energy allocation in this activity had detrimental effects on detoxification, metal elimination or iono-osmoregulation processes. Finally, the lower locomotion of *Gammarus* individuals exposed to both AsV and thermal stress could be linked to a strategy of oxygen consumption reduction. However, the locomotion response was less clear when *G. pulex* was exposed to both Cd and thermal stress. Lannig et al. [84] showed a decline in the mitochondrial capacity to synthesize ATP in the oyster *Crassostrea virginica* when exposed to both Cd and thermal stress. We can hypothesize that this process is also relevant for *Gammarus* exposed to both AsV and thermal stress. Indeed, the competition between AsV and phosphate is temperature-dependent [85]. Thus, this process, in addition to hypoxia driven electron reduction in the mitochondrial chain, could affect the production of ATP. This energetic, oxygen-consuming, metabolism could explain both hyperventilation and mobility reduction in organisms exposed to the combined effects of temperature and metals.

### Synthesis

Based on observed results, we proposed a generalized physiological model of (i) temperature, (ii) cadmium and arsenate individual and combined effects, and (iii) their interactions in *Gammarus pulex* (Fig. 10).

#### Effects of single AsV and Cd exposures

AsV has been known to impede ATP production due to its competition with phosphate. This metal did not hamper the iono−/osmo-regulatory capacity of Na^+^ whereas Cd inhibited the iono−/osmo-regulation because of its ability (i) to compete with Ca^2+,^ and (ii) to bind with Na^+/^K^+^-ATPase. The internal concentrations of AsV and Cd were external concentration-dependent. The mortality of *G. pulex* increased with its internal metal concentration. Both locomotion and ventilation activities decreased when *G. pulex* was exposed to AsV or Cd alone. This decline was amplified when the internal metal concentration of *G. pulex* increased. Such pattern could be a strategy to allocate more energy to within-organism detoxification, elimination or iono-osmoregulation processes in the face of metallic stress. However, we cannot exclude some damages on gills caused by both AsV and Cd even if they would probably be less important for AsV than for Cd, taking into account the already observed (and better known) Cd -related damages.

#### Effects of binary mixture of AsV and Cd

Our results suggested an additive effect of AsV and Cd on the mortality of *G. pulex* for all the tested binary mixture conditions, except As1Cd1 at 10°C. This additive effect of metals in binary mixture was first caused by the individual toxicity of each metal, explaining why toxicity in mixture increased along the AsV (or Cd) gradient for a given concentration of Cd (or AsV). The interactions between the two metals also contributed to this metal additive effect, enhancing or reducing the mixture toxicity on *G. pulex.* The toxicity effects of binary mixtures on haemolymphatic [Cl^−^] increased in gammarids with increasing Cd concentration if compared to the effects observed in gammarids exposed to only AsV. Moreover, mutual synergistic effects of the two metals were especially significant on both locomotion and ventilation. Finally, AsV inhibited the toxicity of the lowest tested Cd concentration because of a “protector effect” on Na^+^ regulatory capacity.

#### Effects of temperature

An increase in temperature is able to cause a depletion of dissolved oxygen in water and the presence of metals (alone or in mixture) can amplify the oxygen depletion effects. Hypoxia in organisms could lead to a reduction of electrons in the mitochondrial chain and affect ATP production. Because arsenate competes with phosphate in respiratory metabolism, ATP production could be more impaired in *G. pulex* exposed to both AsV and warming stress than in individuals exposed to both Cd and warming stress. However, increasing temperature enhanced Cd accumulation but not AsV accumulation in exposed organisms. The increase in both metal accumulation and hypoxia can induce oxidative stress. To survive, *G. pulex* should hyperventilate to satisfy its oxygen demand. Finally, locomotion was reduced to preserve both oxygen and energy. The increase in Cd accumulation - while internal AsV concentration remained stable - with increasing temperature also conducted to more severe depletion in the haemolymphatic [Na^+^] and [Cl^−^] of *G. pulex* exposed to only Cd or to AsV and Cd binary mixture. Finally, osmolality and/or oxygen demand could explain why the rise in temperature increased the mortality of *G. pulex* exposed to a single metal or to a binary mixture.

### Conclusion and Perspectives

To better understand the combined effects of both pollutant cocktails and additional stress, including changes in temperature, it is first necessary to study the individual impact of each toxicant for each temperature. Except the antagonistic effect of the mixture combining the lowest concentrations of AsV and Cd at 10°C, the mode of action of binary mixtures on the mortality of *G. pulex* was considered as additive. In single AsV or Cd exposure, the mortality and internal [AsV] or [Cd] in *G. pulex* were external concentration-dependent. However, these relationships were less clear for binary mixtures. Single cadmium exposure decreased the haemolymphatic [Na^+^] and [Cl^−^] in *G. pulex* while arsenate exposure had no effect on the regulation of [Na^+^] and [Cl^−^]. In binary mixtures, the presence of arsenate seemed to have a “protector” effect on the loss of haemolymphatic Na^+^ due to the cadmium effect. Behavioural activities decreased when *G. pulex* was exposed to AsV or Cd alone. This reduction was amplified in binary mixtures, suggesting a reallocation of energy from behavioural responses to maintenance functions. Finally, the observed physiological and behavioural effects (except ventilation) in *G. pulex* exposed to AsV and/or Cd were exacerbated under the highest (but ecologically realistic) temperature tested. For all the tested conditions, the rise in temperature probably increased the oxygen demand in *G. pulex*, leading to a higher toxicity due to a mismatch between the oxygen demand and the energetic reallocation from ventilatory activity to maintenance functions. These results suggest that in summer or spring, *G. pulex* would be more vulnerable to metals, alone or in mixture. In the same way, at broader temporal scale, climate changes, including global warming, combined with metal contamination of aquatic ecosystems, could accelerate the decline or extinction of *Gammarus* populations, a key species in macrobenthic assemblages, potentially leading to severe alteration in ecosystem functioning. To go further, two main aspects need to be explored. First, we should investigate the mechanisms that can explain the differences in the resulting effects of the interaction between these two metals, depending on their concentrations and the environmental temperature, in particular at low metal concentrations. Second, we would like to examine if the observed effects subsist when gammarids are exposed to mixtures combining metals in a lower range of concentrations, i.e. more likely to be observed in a high number of freshwater ecosystems subjected to anthropogenic pressures. A multimetric approach should be selected combining the measure of both metal concentrations in the model species with several biomarkers and their toxic effects in organisms. Including markers of oxidative stress, at the crossroad of different metabolic pathways, seems to be a good strategy when the toxicant stress impairs gills, leading to energy reallocation and iono-regulation disruption. To better understand the effects of cocktails of pollutants in a context of global change further researches should be also devoted to the evaluation of (i) specific enzymatic activities and (ii) involved metabolic pathway impairments during cellular responses, taking into account temperature fluctuations and extreme event frequency.
